# SLE Complicated With AIHA and Parvovirus B19 Infection Secondary to PRCA: A Case Report

**DOI:** 10.1002/ccr3.73784

**Published:** 2026-10-26

**Authors:** Mingquan Xing, Siqi Zhang, Yanrong Zhang, Xiaoxing Sun, Hongfeng Ge

**Affiliations:** ^1^ Department of Hematology Bozhou People's Hospital Bozhou People's Republic of China; ^2^ Scientific Research and Experiment Center Bozhou People's Hospital Bozhou People's Republic of China

**Keywords:** hemolytic anemia, parvovirus B19, pure red cell aplasia, systemic lupus erythematosus

## Abstract

We report a woman in her 30s with SLE and AIHA who developed parvovirus B19 infection and secondary PRCA. Hemoglobin did not increase significantly after IVIG treatment; her condition improved significantly following the addition of foscarnet, providing a practical treatment option for secondary PRCA.

## Key Clinical Message

Pure red cell aplasia (PRCA) secondary to parvovirus B19 infection was diagnosed after repeated bone marrow aspirations. Diagnosis and treatment were challenging. A favorable response to foscarnet was observed after more than 50 days of ineffective IVIG treatment.

## Graphical abstract

We report the diagnosis and treatment process of a female with SLE and AIHA infected with parvovirus B19 and secondary PRCA. Interestingly, there was no significant increase in hemoglobin after IVIG treatment for more than 50 days; however, the addition of foscarnet showed good efficacy.

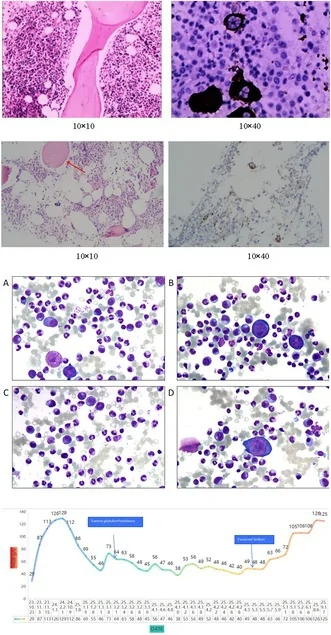

## 1 Introduction

Refractory anemia developing after treatment in patients with SLE and AIHA may initially be attributed to recurrent hemolysis, making PRCA difficult to recognize. Immunosuppressive agents are commonly used to treat PRCA, but their efficacy is limited in virus‐associated PRCA. In this case, no response was observed after prolonged IVIG treatment; however, a marked therapeutic response occurred after foscarnet was administered. We report a rare and easily missed case of parvovirus B19‐associated PRCA in a patient with SLE and AIHA. The main manifestation was refractory anemia, which improved significantly after foscarnet treatment. This case highlights the need to consider PRCA and other differential diagnoses when anemia recurs in patients with SLE and AIHA.

## 2 Case Presentation

### 2.1 Patient Description

A previously healthy woman in her 30s presented to the hospital with fatigue 2 years ago. Routine laboratory examination showed a WBC count of 10.26 × 10^9^/L, HGB of 32.00 g/L, PLT count of 275 × 10^9^/L, IBIL of 100.4 μmol/L, and LDH of 662 U/L. Bone marrow findings were consistent with proliferative anemia (Figure 1). Moreover, anti‐SSA antibody was strongly positive, and antinuclear antibody was positive. Direct and indirect antiglobulin tests were also positive. She was diagnosed with systemic lupus erythematosus (SLE) with autoimmune hemolytic anemia (AIHA). After treatment with corticosteroids, rituximab, methotrexate, and hydroxychloroquine, her condition gradually improved. The corticosteroid dose was reduced and then maintained.

**FIGURE 1:**
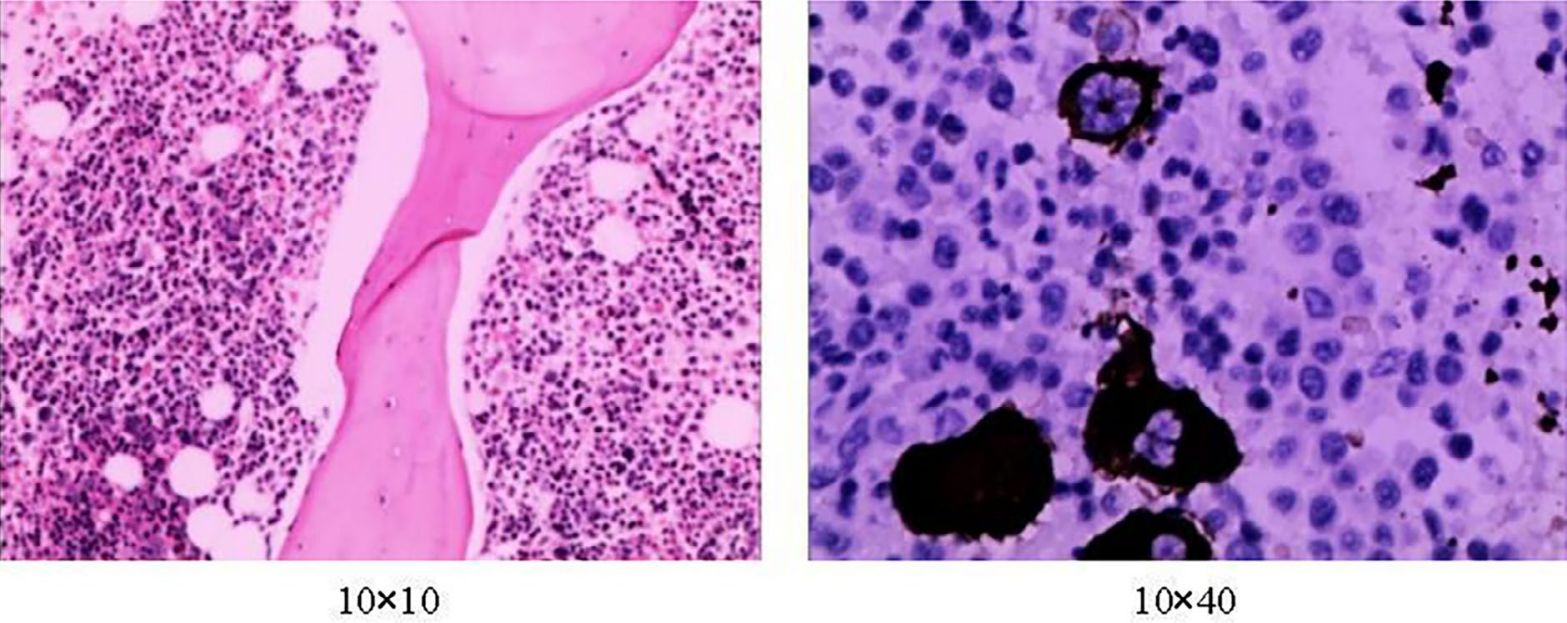
Bone marrow pathology exhibited proliferative anemia.

In January 2025, anemia recurred. The reticulocyte (Ret) count and HGB level decreased, with a poor response to blood transfusion. Upon transfer to a provincial hospital, bone marrow aspiration and additional examinations were performed after iron, folic acid, and vitamin B12 deficiencies, gastrointestinal bleeding, menorrhagia, hemorrhoids, inflammation, and hypothyroidism had been excluded. Bone marrow pathology showed giant immature erythroid cells, with megakaryocytes present (Figure 2). Bone marrow morphology also showed giant primitive erythroid cells (Figure 3), supporting the diagnosis of pure red cell aplasia (PRCA). Immunosuppressive therapy was ineffective, and the HGB level continued to decline. The patient was subsequently evaluated at the Institute of Hematology and Blood Diseases Hospital, Chinese Academy of Medical Sciences, where repeated bone marrow aspiration again supported the diagnosis of PRCA. After unsuccessful immunosuppressive therapy, parvovirus B19 (B19V) DNA testing was positive (Figure S1).

**FIGURE 2:**
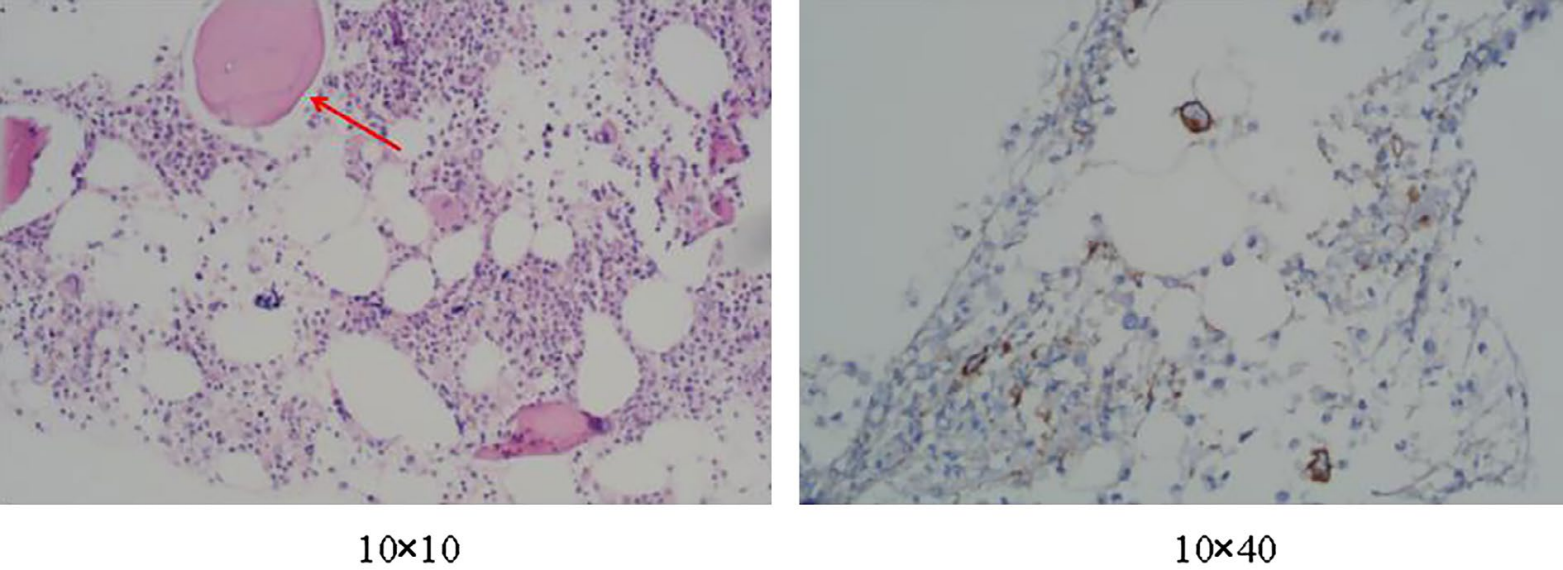
Bone marrow pathology showed active proliferation of hematopoietic tissue, increased proportion of granulocytes, decreased erythroid proliferation, giant proerythroblasts were seen and visible megakaryocytes.

**FIGURE 3:**
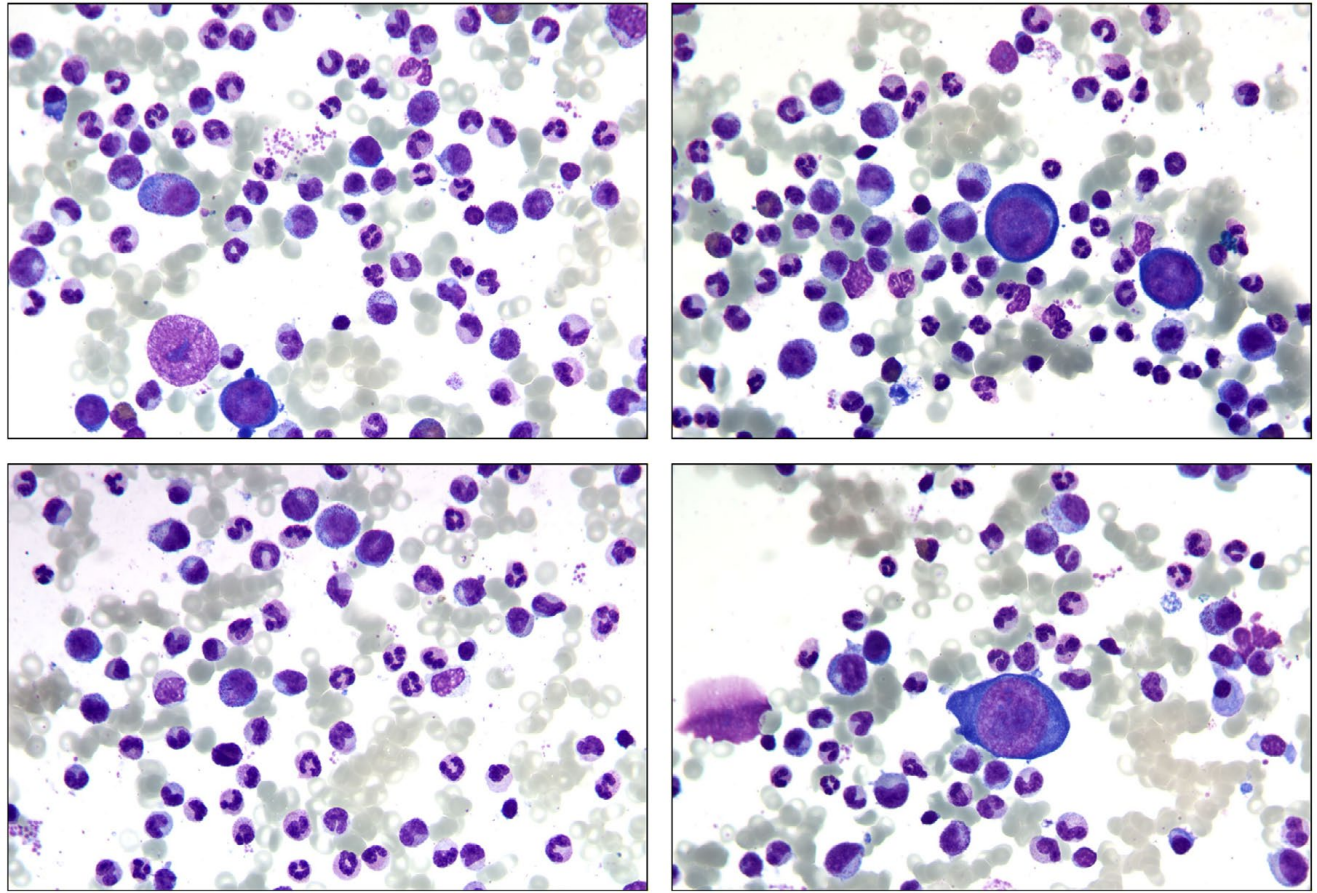
Bone marrow aspirate smear showing decreased erythroid precursors and a giant pronormoblast (Wright‐Giemsa stain, × 1000).

### 2.2 Differential Diagnosis and Diagnostic Reasoning

The patient had SLE and AIHA and had received long‐term immunosuppressive therapy. When the HGB level decreased, recurrent or aggravated hemolysis related to SLE was initially considered, and the dose of immunosuppressive therapy was increased; PRCA was not initially considered. Because the clinical response remained unsatisfactory, repeated bone marrow aspiration was performed and supported the diagnosis of PRCA. The reduced reticulocyte (Ret) count and HGB level, together with the presence of giant immature erythroid cells on bone marrow pathology and morphology, were helpful for diagnosing PRCA [1]. PRCA should be differentiated from aplastic anemia (AA), which typically presents with multilineage hypoplasia. It should also be differentiated from myelofibrosis and myelosuppression after radiotherapy or chemotherapy; these conditions can be distinguished through bone marrow biopsy and clinical history. After PRCA was diagnosed, primary and secondary causes were investigated. Potential secondary causes included thymoma, drug‐related PRCA, B‐cell dyscrasia‐related PRCA [2], transplantation‐related PRCA, and other infections. Parvovirus B19 was ultimately identified as the etiologic agent in this patient.

### 2.3 Investigations, Treatment, and Follow‐Up

PRCA is commonly treated with immunosuppressive agents. Because this case was associated with parvovirus B19 infection, the response to immunosuppressive therapy was unsatisfactory. Corticosteroid doses were reduced (prednisone acetate tablets, initially 15 mg QD and then reduced to 5 mg QOD), and immunosuppressive therapy was adjusted (cyclosporine 150 mg BID, then reduced to 50 mg BID and discontinued on 14 April 2025), together with intravenous immunoglobulin (IVIG). The initial IVIG dose was 10 g/day for 3 days and was later changed to 10 g once every 5 days. After 20 days of treatment, HGB did not increase significantly. The IVIG dose was adjusted to 20 g/day for 5 days, without a favorable response. After intermittent IVIG administration (12.5 g on 16 April, 15 g on 23 April, and 20 g on 29 April 2025), the HGB level remained low. Foscarnet was started on 3 May 2025. HGB gradually increased after 3 days and normalized and stabilized after 10 days (Figure 4); parvovirus B19 DNA subsequently became negative. The patient has been followed to the present. Parvovirus B19 testing has remained negative (Figure S2), and HGB has remained within the normal range. She has returned to daily activities and work.

**FIGURE 4:**
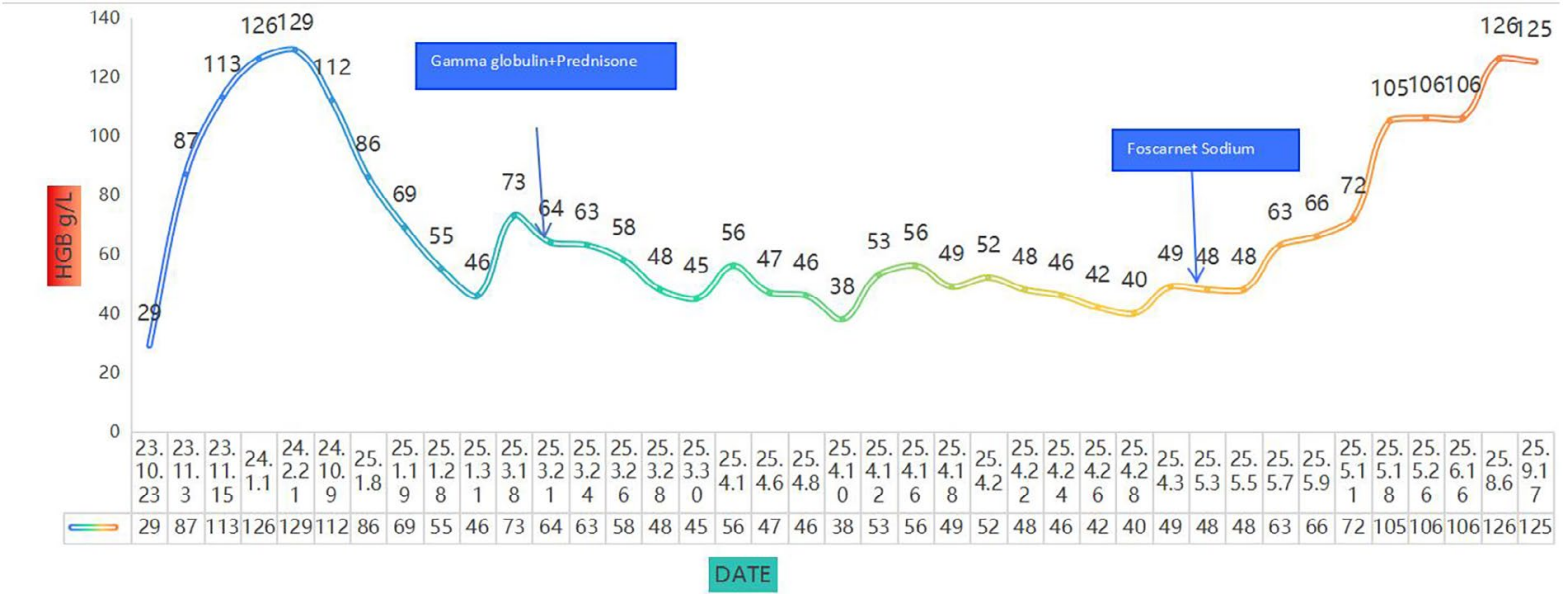
The trend of hemoglobin changes during PRCA treatment.

Parvovirus B19‐associated PRCA is uncommon, and the available PubMed literature consists predominantly of case reports. The findings of the literature review are summarized in Table 1. Most reported patients had undergone organ transplantation, and improvement was generally observed after IVIG treatment. However, the present patient showed a poor response to IVIG treatment.

**TABLE 1:** Summary of demographics, underlying disease treatment, and prognosis of parvovirus B19 secondary PRCA published in English in PubMed since 1990.

Author	Year	Sex/age	Location	Underlying diseases	PMID	Treatment	Outcome
Lindahl JP et al.	2022	M/38	Norway	pancreas transplantation	35,372,384	IVIG	Stable
Eoin et al.	2015	M/11	Ireland	heart transplantion	24,555,951	IVIG	Stable
Santokh et al.	2014	F/30	India	Healthy person	24,959,472	PSL	Stable
Chun et al.	2012	F/29	Australian	heart transplantation	23,256,205	IVIG	Stable
Arundathi et al.	2011	M/30	Oman	kidney transplantation	21,969,904	IVIG	Stable
Hiroto et al.	2011	F/36	Japan	Healthy person	20,820,839	No treat	Stable
Hiroto et al.	2011	F/55	Japan	small‐lympho‐cytic lymphoma	20,820,839	PSL	Stable
Eun et al.	2010	F/57	Korea	liver transplantation	21,157,144	IVIG	Stable
Betul et al.	2009	M/10	Turkey	T‐cell immunodeficiency	19,322,736	IVIG	NS
Haruko et al.	2007	F/82	Japan	SLE	17,028,860	PSL	death
Liang et al.	2007	M/38	China	liver transplantation	17,461,508	IVIG	Stable
Poj et al.	2006	F/51	Thailand	HIV	16,313,965	IVIG	Stable
Prashant et al.	2006	M/63	India	CLL	17,178,664	No treat	Stable
Prashant et al.	2006	M/12	India	NF‐1	17,178,664	No treat	Stable
Mihail et al.	2005	M/14	India	cadaveric renal transplant	16,269,055	IVIG	Stable
Hajime et al.	2003	F/28	Japan	UC	14,627,362	IVIG	Stable
Negami et al.	1994	F/38	Japan	IDA	8,065,019	No treat	Stable
Niitsu et al.	1990	F/14	Japan	allo‐HSCT	2,174,087	IVIG	Stable
This case	2025	F/38	China	SLE		IVIG+Foscarnet Sodium	Stable

Abbreviations: allo‐HSCT, allogeneic hematopoietic stem cell transplantation; CLL, chronic lymphocytic leukemia; HIV, human immunodeficiency virus; IDA, iron deficiency anemia; IVIG, intravenous immunoglobulin; NF‐1, neurofibromatosis type‐1; NS, not specific; PSL, prednisolone; SLE, systemic lupus erythematosus; UC, ulcerative colitis.

Marked improvement was observed after foscarnet therapy. The potential synergistic mechanism warrants further investigation.

## 3 Discussion

PRCA is a rare bone marrow failure disorder characterized primarily by normocytic normochromic anemia and a decreased Ret count. Although erythroid precursor cells in the bone marrow are often absent or markedly reduced, granulocytes and megakaryocytes are usually preserved [3]. The pathogenesis of PRCA is complex, and the disorder can be classified as congenital or acquired. Major causes of acquired PRCA include drugs, viral infections, thymoma, and hematopoietic stem cell transplantation [4]. Among these, thymoma‐related PRCA is the most common cause of secondary PRCA. PRCA with an identifiable cause is classified as secondary PRCA, whereas primary PRCA has no known cause. It occurs worldwide without clear regional differences. SLE is an autoimmune disease that can affect multiple organs and systems. It primarily affects young women and is characterized by immune activation. When SLE involves the hematologic system, red blood cells or other blood cell lineages may be reduced.

Therefore, anemia is a common hematologic manifestation of SLE [5]. Abnormal immune activation can cause AIHA, and hemolysis is a common complication of SLE [6]. Patients with hemolysis may respond poorly to blood transfusion because of autoantibody production. However, PRCA may not be considered in patients with SLE and AIHA, resulting in a relatively high risk of missed diagnosis. In the present patient, autoimmune hemolytic anemia was the initial manifestation.

After bone marrow aspiration, SLE and AIHA were diagnosed. Her condition stabilized after corticosteroid and other treatments. However, the HGB level later continued to decline, and the response to transfusion was poor. Treatment of the underlying disease was intensified to control hemolysis, but the HGB level continued to decrease. Ultimately, repeated bone marrow aspirations were performed at different sites, and PRCA was diagnosed. Current treatment for PRCA includes immunosuppressants such as cyclosporine and sirolimus [3]. The response to immunosuppressants in this patient was poor, and subsequent therapeutic attempts were ineffective. Further evaluation identified parvovirus B19 infection. Because no specific treatment is established for this virus, high‐dose IVIG is commonly combined with conventional low‐dose immunosuppressive therapy [7]. Despite more than 50 days of treatment, the response remained poor. After foscarnet was added, HGB increased significantly and stabilized within the normal range. Parvovirus B19 DNA gradually became negative and has remained negative during follow‐up.

Parvovirus B19 is a common viral cause of PRCA [8]. Other viral causes include EBV and hepatitis C virus. Parvovirus B19 may remain latent in the general population and is more likely to cause infection in immunocompromised patients, including organ transplant recipients. It can cause myocarditis, central nervous system involvement, and gastrointestinal disorders [9]; some infected individuals may develop PRCA. Currently, no specific antiviral drug is established for parvovirus B19 infection. Treatment includes supportive care, immunoglobulin, corticosteroids, and other symptomatic measures [10]. IVIG can be effective for parvovirus B19‐related PRCA. In the present case, however, no significant improvement was observed after continuous IVIG administration for more than 50 days. After foscarnet therapy, the HGB level steadily increased, normalized within a short period, and remained stable. Parvovirus B19 DNA also became negative. Foscarnet is used primarily for antiviral treatment of cytomegalovirus in immunocompromised patients, including patients with AIDS [11]. Foscarnet selectively inhibits the pyrophosphate‐binding site of virus‐specific DNA polymerase, thereby exerting antiviral activity. Its use has also been reported for parvovirus B19 infection [12].

## 4 Conclusion

(1) In patients with SLE and AIHA who respond poorly to immunotherapy and blood transfusion, PRCA should be considered. (2) In patients with PRCA, the underlying cause should be identified; immunosuppressive therapy may be ineffective in virus‐associated PRCA. (3) When IVIG alone is ineffective, antiviral therapy may be considered in combination with IVIG. Reported options include the nucleotide analog cidofovir, its lipid conjugate brincidofovir, and foscarnet.

## Author Contributions

**Mingquan Xing:** investigation, conceptualization, formal analysis, writing – original draft, writing – review and editing, funding acquisition. **Siqi Zhang:** investigation, formal analysis. **Yanrong Zhang:** investigation. **Xiaoxing Sun:** supervision, project administration. **Hongfeng Ge:** supervision, project administration.

## Funding

This work was supported by the Scientific Research Foundation of Anhui Medical University (No. 2023xkj090) and the Key Research and Development Projects of the Bozhou Science and Technology Bureau (No. bzzc2024011).

## Consent

Written informed consent was obtained from the patient to publish this report in accordance with the journal's patient consent policy.

## Conflicts of Interest

The authors declare no conflicts of interest.

## Supporting information


**Figure S1:** Parvovirus B19 (B19V) DNA was positive.
**Figure S2:** The patient's parvovirus B19 has turned negative.

## Data Availability

Data sharing not applicable to this article as no datasets were generated or analysed during the current study.
