# Supplementary material for: SLE Complicated With AIHA and Parvovirus B19 Infection Secondary to PRCA: A Case Report
Source: Clin Case Rep. 2026 Oct 26;14(11):e73784. doi: 10.1002/ccr3.73784 (PMC13698829; doi:10.1002/ccr3.73784)
Supplement: Supplementary file 1 [file CCR3-14-e73784-s001.doc]

Clinical real images

Parvovirus B19 (B19V) DNA was positive.

The patient's parvovirus B19 has turned negative.
